# Immunological aspects of necrotizing enterocolitis models: a review

**DOI:** 10.3389/fimmu.2024.1434281

**Published:** 2024-07-22

**Authors:** Laura Blum, Deirdre Vincent, Michael Boettcher, Jasmin Knopf

**Affiliations:** Department of Pediatric Surgery, University Medical Center Mannheim, University Heidelberg, Mannheim, Germany

**Keywords:** necrotizing enterocolitis, immune system, animal models, organoids, immune cells

## Abstract

Necrotizing enterocolitis (NEC) is one of the most devasting diseases affecting preterm neonates. However, despite a lot of research, NEC’s pathogenesis remains unclear. It is known that the pathogenesis is a multifactorial process, including (1) a pathological microbiome with abnormal bacterial colonization, (2) an immature immune system, (3) enteral feeding, (3) an impairment of microcirculation, and (4) possibly ischemia-reperfusion damage to the intestine. Overall, the immaturity of the mucosal barrier and the increased expression of Toll-like receptor 4 (TLR4) within the intestinal epithelium result in an intestinal hyperinflammation reaction. Concurrently, a deficiency in counter-regulatory mediators can be seen. The sum of these processes can ultimately result in intestinal necrosis leading to very high mortality rates of the affected neonates. In the last decade no substantial advances in the treatment of NEC have been made. Thus, NEC animal models as well as *in vitro* models have been employed to better understand NEC’s pathogenesis on a cellular and molecular level. This review will highlight the different models currently in use to study immunological aspects of NEC.

## 1 Introduction

Out of all gastrointestinal disease affecting premature infants, necrotizing enterocolitis (NEC) is one of the most devasting, resulting in major mortality rates in neonatal intensive care units (NICUs). More specifically, NEC is considered to be the leading cause of death in preterm infants born before 29 weeks of gestation at 28–60 days postpartum. Neonates most affected by the disease are those with a birth weight of less than 1500 g, where the incidence lies between 3-15% (1–3).

Despite intensive research, NEC’s pathogenesis has remained unclear. However, research suggests the process to be multifactorial. One key player seemingly involved in the development of NEC is the immature immune system of preterm neonates, which has been identified to lead to an uncontrolled, excessive immune response and sepsis reaction after initial pathogen contact (4). For NEC onset in particular, the combination of (1) an immature gastrointestinal tract with (2) a pathological microbiome involving an abnormal bacterial colonization (5), combined with (3) an immature immune system is hypothesized to result in an increased expression of TLR4 within the intestinal epithelium. This in turn has been shown to lead to an excessive secretion of pro-inflammatory mediators (6). Simultaneously, due to the immaturity of the immune system in neonates developing NEC, a deficiency in counter-regulatory mediators has been observed, which ultimately results in an inflammatory milieu. Moreover, increased apoptosis of enterocytes and impaired healing of the affected mucosa, combined with an increased intestinal permeability and a reduced blood flow within the intestinal tissue lead to ischemia, which contributes to tissue damage and necrosis (5, 7, 8).

### 1.1 Risk factors

Because NEC’s pathogenesis is proposed to be of multifactorial nature, certain risk factors have been identified, which favor the formation of NEC. The main risk factors are as follows:

#### 1.1.1 Prematurity

Clinical data suggests that NEC is associated with prematurity of the affected neonate. Although the intestinal tract completes its development early during embryogenesis, it has not completed maturing until term gestation (9–11). In fact, prematurity is the most critical risk factor for NEC development, as preterm born neonates have an immature intestinal barrier and immune system. Moreover, neonates born preterm tend to exhibit poor gut motility making them particularly susceptible to bacterial translocation (12, 13). As preterm neonates express higher levels of TLR4 in the intestine compared to term-born neonates, a dysregulation of the immune system may lead to extensive mucosal injury due to an intestinal hyperinflammation reaction and enterocyte apoptosis, as observed in patients with NEC (14, 15).

#### 1.1.2 Dysbiosis

It is known that the intestinal microfilm plays an essential role in gut homeostasis and protection. Therefore, dysbiosis of such has been shown to be an important factor in the pathogenesis of NEC. The origin and development of the neonate’s gut microbiome are still under investigation. However, there is evidence that the microbiome of neonates is determined by (1) the mother’s microbiome, (2) the mode of delivery, (3) enteral nutrition, as well as (4) the postnatal environment (16). With regards to NEC development, studies have demonstrated a dysbiotic shift in the gut microbiota prior to the onset of NEC, with an increase in Proteobacteria and a decrease in Firmicutes and Bacteroidetes (17). In line with these findings, the administration of antibiotics, which is known to influence the gut’s microbiome negatively, has also been shown to increase the risk for NEC development (18). Even though, the exact mechanism by which the microbiome influences gut health and how this might lead to NEC onset is not well understood, it is believed that the combination of (1) an immature gut, (2) limited absorption and digestive capacity, (3) a dysbiotic microbiome, and (4) delayed intestinal motility results in an intestinal environment that is characterized by bacterial overgrowth and fermentation (19) as well as an impaired mucosal barrier (20) in preterm infants.

#### 1.1.3 Formula feeding

Enteral feeding has been shown to be a critical risk factor for the development of NEC. Breast milk contains different components, which are important for the immune system development of the preterms. These immunomodulating components are lacking or present in lower quantities in formula milk. Consequently, infants who are formula-fed may have a higher susceptibility to NEC compared to those who are breastfed (21).

#### 1.1.4 Maternal conditions

The maternal-fetal connection plays also a crucial role in NEC development and can influence the NEC risk and severity. Studied have found an association between maternal hypertension and preeclampsia. This can lead to placental abruption and fetal hypoxia, both of which increase the risk of NEC. But bacterial infections *in utero* play a role in the development of NEC (22, 23).

### 1.2 Prevention of NEC

Breast milk contains various bioactive components such as Immunoglobulins (Ig), cytokines or antimicrobial factors, which play a crucial role in the infant’s immune system development and defense against infections. Especially maternal IgA has shown to shapes the host-microbiota relationship of preterm infants (24). Breast milk not only include IgA, but also human milk oligosaccharides (HMOs). HMOs are structurally complex glycans and abundant in milk with different concentrations varying based on the stage of lactation. They are metabolized by different intestinal bacteria and play a beneficial role in preventing NEC. They enhance host defense, modulate immune cell function and improve the integrity of the intestinal barrier (25–27).

Aryl hydrocarbons receptors (AHR) are widely expressed in immune cells and non-immune cells. Their regulation is correlated with maintenance of homeostasis. AHRs also regulate the intestinal barrier and immune cells. Therefore, they possess anti-inflammatory properties. A deletion of AHRs exacerbated intestinal inflammation in mice. It was also reported that an increased expression of AHRs can reduce the secretion of pro-inflammatory cytokines in NEC and thus the NEC-induced intestinal damage. The AHR ligand indole-3-carbinol (I3C) as well as breast milk can activate AHRs. I3C are derived from different vegetables and can be used for dietary supplementation against NEC. A study showed that the activation of AHRs during pregnancy can protect newborns against NEC. This protective mechanism can also reduce TLR4 signaling (28, 29).

Blood perfusion studies cannot be used for the prevention but for the early detection of NEC by focusing on the blood flow dynamics in the intestine. This is helpful to indicate the severity of NEC or monitor the progress since impaired blood perfusion is a significant factor contributing to NEC pathogenesis. There are three methods commonly employed to measure the blood flow: Doppler ultrasound, near-infrared spectroscopy and laser-doppler flowmetry (30–32).

Another way to prevent NEC could be the inhibition of TLR4, as it plays an important role in the pathogenesis of NEC. Inhibition of the receptor could reduce the multifactorial inflammatory process. Several inhibitors are already known, but none is used for clinical treatments yet. Inhibitors can be peptides (33), oligosaccharides (34) or small molecules (35) but amniotic fluid (36) can also inhibit TLR4.

## 2 NEC models

To date, preclinical models studying NEC have focused on animal models in order to improve the understanding of the disease on a cellular and molecular level (16, 37, 38). Animal models over the decades have employed mice, rats, quails, rabbits, pigs, and baboons. Out of these research subjects, mice, rats and piglets have proven the most widely used animal to study NEC. However, as each model has advantages as well as disadvantages results obtained solely from animal models can only mimic NEC (37) and findings often prove difficult to translate to human subjects. As such, other non-animal NEC models have been developed like organoids. Organoids are derived from cultured intestinal and pluripotent stem cells (39, 40) and are self-organized 3D-structures that mimic the properties and physiological characteristics of the intestinal epithelium (41). Therefore, there is the anticipation that organoids may close the gap between animal and human NEC models, aiding in the translation from bench-to-beside.

In this review, we have focused on current immunological aspects involved in the NEC pathogenesis while identifying the different models employed to study NEC.

### 2.1 Animal models for NEC research

Despite the development of different animal NEC-models, with different methods to induce NEC, no one model is superior, as each model has their advantages and disadvantages (**Table 1**). The most common model is the Hypoxia Hypothermia Formula model (HHF). This model can be used to study combined effects as it closely mimics multiple risk factors for NEC. For this, the pub is exposed to brief periods of hypoxia and hypothermia combined with formula feedings for several days. The Hypoxia Formula model (HF) is a two-component model without hypothermia to induce NEC. This model can offer more consisting results in NEC manifestation, as is has fewer variables for NEC induction. The Formula Feeding model (FF) can be used to study the influence of formula diet, the main risk factor of NEC. The models can be modified and improve by adding components such as LPS or other bacterial components to the formula diet to support the NEC induction in the models and increase the severity in the animals.

**Table 1:** Advantages and disadvantages of different methods to induce NEC in animals.

Model	Advantages	Disadvantages	References
HHF	• Multiple-hit approach: disrupts the protective mucosal barrier, alters the microbiota environment for dysbiosis • Uses known clinical risk factors	• Animals develop intestinal damage at different time points • Gavage feeding of newborn mice • Comparison of histology and enzyme ontogeny shows that newborn mice resemble human fetuses at 16 weeks’ gestation, which is developmentally less mature than the age at which humans develop NEC • Hypoxia and hypothermia are big stressors	(16, 37, 42)
HF	• Less stressful, hypothermia is omitted • Uses known clinical risk factors	• Gavage feeding of newborn mice • Comparison of histology and enzyme ontogeny shows that newborn mice resemble human fetuses at 16 weeks’ gestation, which is developmentally less mature than the age at which humans develop NEC • Hypoxia is a big stressor • Loss of hypothermia influence	(16, 37, 42)
FF	• Good to study different components of formula or hyperosmotic formula	• Gavage feeding of newborn mice • Comparison of histology and enzyme ontogeny shows that newborn mice resemble human fetuses at 16 weeks’ gestation, which is developmentally less mature than the age at which humans develop NEC	(16, 37, 42)
TNBS	• Non-specific immunological stimulant • Accurate mapping of the time course of intestinal damage from the time point of TNBS administration • The induced cellular inflammatory response resembles surgically harvested tissue samples from NEC • Requires the presence of intestinal microflora • Administration of weight-normalized dose may facilitate the study of developmental aspects of NEC • Murine neonatal TNBS-mediated intestinal injury activates transcriptional profiles that strongly resemble human NEC. • Good for macrophage research	• Different inflammatory processes in neonatal and adult mice • Inflammatory induction mechanism is not clear	(43, 44)
PCD	• For mature Paneth cells, mice must be around 14-16 days old (no neonatal mice) • No gavage feeding	• Focus on Paneth cell as NEC -like inflammation trigger.	(42, 45, 46)
DSS	• Absence of stressors like hypoxia and/or hypothermia • Short time until first mucosal change after DSS application • Humoral and cellular immune response	• No NEC, just NEC-like-mucosal-tissue injury	(47)

HHF, hypoxia-hypothermia-formula feeding; HF, hypoxia-formula feeding; FF, formula feeding; TNBS, trinitrobenzene sulfonate; PCD, paneth cell disruption; DSS, dextran sodium sulfate.

Nevertheless, translation of findings from NEC animal models to human neonates remains problematic. This may be due to differences in the development of the small intestine (48), as well as differences of the animal’s immune system in comparison to neonates (49). Thus, only partial aspects of the NEC disease process can be examined using NEC animal models. The individual models and their use are discussed in more detail in section 3.

## 3 Immunological aspects of different animal models for NEC

One of the major risk factors for NEC development is the immaturity of the immune system of the preterm born neonate. Specifically, an array of different cells and mediators of the innate and adaptive immune systems have been implicated with NEC development (49). On one hand, the innate immune system acts as a first line defense against potential pathogens. Not only does it make up a physical barrier against microbial entry, but it also mobilizes various types of immune cells and mediators for a rapid response against infectious agents. On the other hand, the adaptive immune system responds to highly specific antigens via different T cells and B cells.

**Figure 1:**
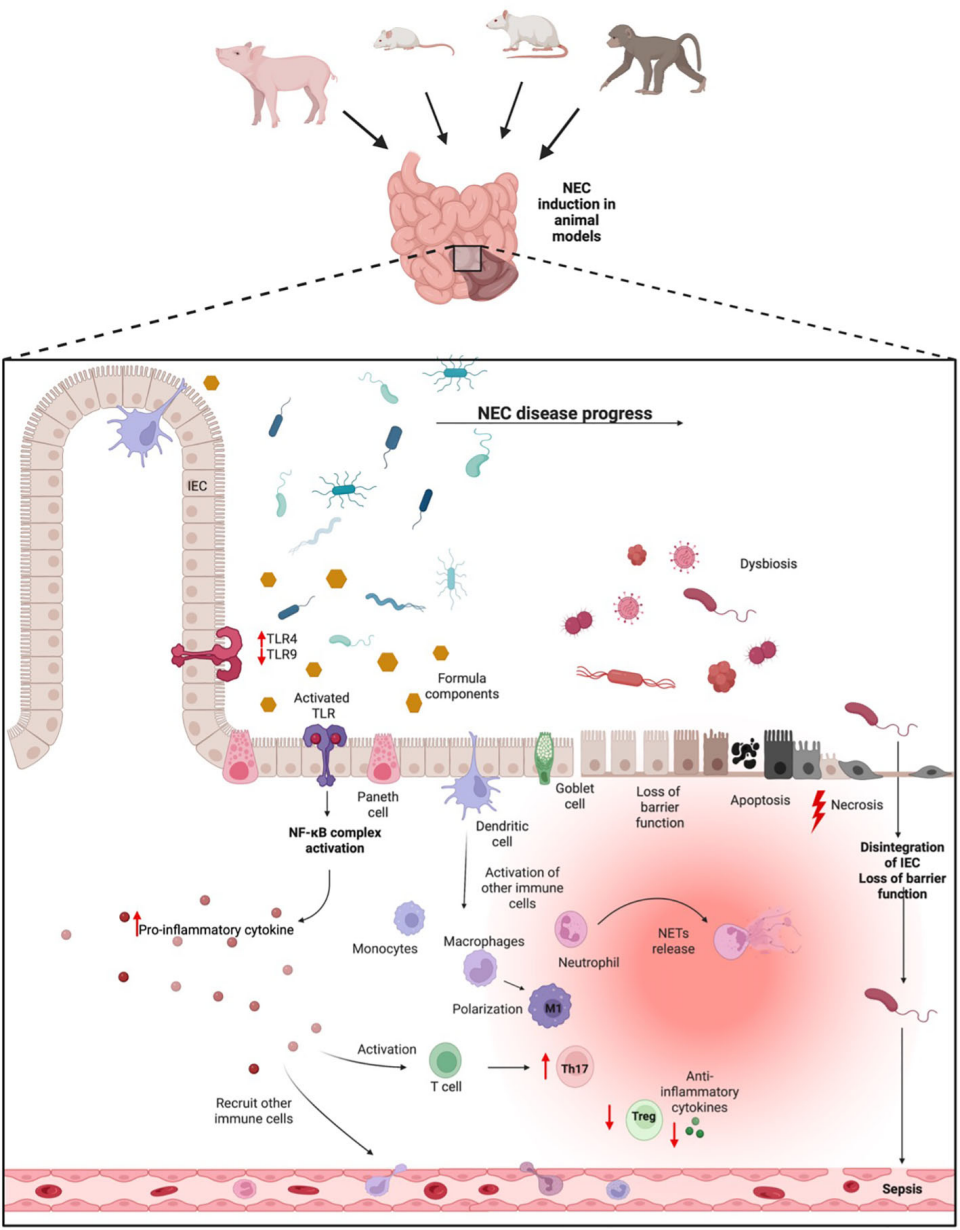
NEC pathogenesis. In animal NEC models, NEC can be induced via different methods. In neonates that go on to develop NEC, a shift of the microbiome into a dysbiosis during the inflammatory progress takes place. This dysbiosis can be pronounced through formula feeding, which together might activate the TLR dependent inflammatory process. Activation of the TLR pathway leads to the release of pro-inflammatory cytokines, which in turn recruit immune cells from blood. Concurrently, the inflammatory process results in a loss of epithelial barrier function, apoptosis, and necrosis of intestinal tissue, resulting in potential bacterial penetration from the gut into the blood, ultimately leading to sepsis. IEC, Intestinal epithelial cells; TLR, Toll-like-receptor; Treg, T-regulatory cell; M1, pro-inflammatory Macrophages.

So far, with respect to the immune system and NEC development, various mouse lines, rats, piglets, and baboons have been employed to study different aspects of NEC pathogenesis. A summary regarding the various animal models and their immunological features can be found in the following paragraphs and **Figure 1**. Additionally, an overview of the different experimental setups of the models used to study the immunological aspects can be found in the **Supplementary Material**.

### 3.1 Hypoxia hypothermia formula (HHF)

The first animal model for NEC research was described in 1974 (50). After birth, rat pubs were contaminated by receiving an oral saline suspension containing Klebsiella organisms shortly after birth. Additionally, mothers were contaminated transvaginally using a plastic tube containing a Klebsiella solution 24 hours before the expected birth. Hypoxia was produced using a plastic bag. Animals were enclosed in a plastic bag once daily for 3-5 min until they were flaccid, cyanotic, and gasping. This model was further intensified by hypothermia. In this process, the pups were cooled, resulting in lower intestinal blood flow and ischemia. This model was therefore called the HHF (Hypoxia Hypothermia Formula) model. Years later, Caplan et al. introduced bacterial pathogens into the formula given to newborn rats. This resulted in a NEC-like intestinal injury, highlighting the critical role of pathogenic bacterial colonization in the development of NEC (51). The HHF model was also adapted to mice with different variations of the bacterial pathogens administered.

#### 3.1.1 Cells of the innate immune system studied with the HHF model

To examine the role of macrophages in NEC, MohanKumar et al. used the HHF mouse model in 2016 to investigate Smad7, which plays a role in regulating inflammation and immune response. The researchers aimed to better understand how macrophages contribute to the development and progression of NEC, particularly through the modulation of Smad7 signaling pathways. They showed that the activation of macrophages resulted in increased Smad7 expression during NEC, especially in areas with severe intestinal tissue damage and high bacterial load. Thereby, increased expression of Smad7 resulted in suppressed transforming growth factor ß (TGF-ß) signaling and increased NF-κB and cytokine production. This in turn was shown to promote the inflammatory processes observed in NEC (43).

In 2021, another study conducted by Xia et al. focused on the role of intestinal macrophages and TNFα. In this study, which also made use of the HHF mouse model, an increased number of M1 macrophages was found in NEC tissue. This goes hand in hand with an increased expression of CD68, iNOS, and TNFα and a suppressed c-kit expression through TNFα-mediated upregulation of miR-222. This leads to an increased inflammatory response. What is of particular interest is the fact that the inhibition of M1 macrophages, TNFα or miR-222 during the early phase of NEC development could be a potential therapeutic strategy for the treatment of NEC (52).

#### 3.1.2 Pattern recognition receptors (PRR) studied with the HHF model

The HHF model is one of the most commonly used animal models to study toll-like receptors (TLR). Jilling et al. focused on the role of bacteria and TLR4 in a rat and mouse HHF model. Overall, they were able to demonstrate that bacteria play a crucial role in the pathogenesis of NEC and that an increased expression of TLR4 in intestinal epithelium seemed essential for NEC development (53).

Following these results, Le Mandat Schultz et al. evaluated the specific intestinal epithelial expression of TLR using a HHF rat model. Their study suggested an interaction between TLR and intraluminal bacteria and/or bacterial products, in that TLR4 and TLR2 were activated and abnormally upregulated on intestinal epithelial cells. This ultimately lead to the expression of other inflammatory mediators and NEC (54).

A HHF rat model was also employed to examine the potentially protective effect of glutamine and its possible association with TLR4 and TLR2 in NEC. Based on the knowledge that TLR is increasingly expressed in the intestinal mucosa during NEC, this group showed that glutamine significantly reduced mucosal damage while suppressing the expression of TLR2 and TLR4 (55).

Using a HHF mouse model, mRNA expression and interplay of TLR4 and TLR9 in intestinal tissue were investigated (56). Interestingly, the results showed that TLR4 expression was significantly higher whereas TLR9 expression was significantly lower in mice with induced NEC. This counterplay seems to play a crucial role during the inflammatory process observed during NEC.

A newer study examined the role of necroptosis as intestinal epithelial cell death and consecutive barrier dysfunction has been shown to facilitate NEC pathogenesis. Overall, they were able to show that TLR4 expression, as well as necroptotic proteins like mixed lineage kinase domain-like (MLKL) and receptor interacting protein kinase 1 and 3 (RIPK1, RIPK3) were significantly upregulated in NEC models (57).

In the same year, Huang et al. focused on MD2 inhibition as a potential way to counter the excessive expression of TLR4 during NEC. Their results suggest that MD2 might be a potential treatment for reducing the incidence of NEC, as it had an inhibiting effect on TLR4 expression in the animal model (58).

One of the newest studies investigated astaxanthin as a potential therapeutic measure to attenuate NEC. Astaxanthin is a naturally occurring carotenoid pigment with anti-inflammatory effects and antioxidant properties. Astaxanthin improved gut tissue health in NEC rats by reducing inflammation, oxidative stress, and apoptosis. In doing so, it increased NOD2 activity and suppressed TLR4 signaling. Overall, astaxanthin showed a potential as a therapeutic agent to alleviate NEC-related complications (59).

#### 3.1.3 Inflammatory mediators studied with the HHF model

Inflammatory mediators are produced and secreted by various cells of the immune system in response to infections, injury, or other stimuli. They play an important role in initiating and/or resolving inflammations in the human body. They can recruit other immune cells and coordinate the body´s response against an infection or after an injury.

IL-6, as a pro-inflammatory mediator, plays a significant role during NEC pathogenesis. Yarci et al. studied this cytokine using the HFF model in newborn rats in 2021. They were able to report that treatment with tocilizumab, an antibody that blocks IL-6 mediated signaling, led to an decreased expression of the inflammatory profile during NEC (60).

Another study investigated the role of IL-10, an anti-inflammatory mediator, that has been shown to play a role in epithelial integrity and modulation of the immune system. Therefore, its potential protective role in NEC was studied using newborn mice and rats. The study demonstrated that severe morphological and histological changes occurred in mice lacking IL-10. These were manifested by increased apoptosis of the epithelium, decreased localization of junctional adhesion molecule-1, and increased expression of inducible nitric oxide synthase in the intestine. Thus, IL-10 can be presumed to play a protective role in the pathogenesis of NEC (61).

To examine the effect of TNFα on the inflammatory response during NEC, immunoneutralizing TNFα studies using selective antibodies were performed (62). Firstly, Etanercept, a recombinant dimer of the TNFα-receptor protein and therefore a TNFα antagonist, was examined. The results of the study suggest that Etanercept has a positive effect on antioxidative enzymes in tissues and, in addition, attenuated intestinal tissue damage by reducing inflammation. (63).

Second, Infliximab, an inhibitor of TNFα, was investigated as a potential treatment for NEC. As Infliximab binds to TNFα with a high affinity and specificity, administration of Infliximab in the rat model resulted in a reduction of intestinal damage and apoptosis. In addition, Infliximab showed intestinal protective effects (64).

To assess the role of NF-κB in NEC development, De Plaen et al. studied the selective inhibition of NF-κB in the neonatal rat intestine in 2007. Three different objectives were investigated in this study. First, the developmental regulation of NF-κB activation and inhibitory proteins IB in the neonatal rat intestine. Second, the alteration of NF-kB activity in experimental neonatal NEC. Third, the effects of selective NF-kB inhibition on NEC incidence. Overall, the authors were able to demonstrate that NF-κB is persistently active during NEC in the rat model and that this overactivity could have a detrimental effect on intestinal tissue (65).

With respect to the previously summarized study by DePlaen et al., a more recent study focused on the inhibition of NF-κB activation. The study shows that NF-kB activation in Ly6c^+^ monocytes plays a critical role in the promoting intestinal inflammation and therefore in the development of NEC. Deletion of NF-κB in lysozyme M-expressing cells prevented activation, recruitment, and differentiation of monocytes in the neonatal gut, resulting in improved survival and reduced severity of NEC in mice. These results suggest that targeting the early recruitment of monocytes in the intestine may be a promising strategy to prevent NEC-related intestinal injury (66).

Lu et al. investigated platelet-activating factor (PAF) and the plasma form of platelet-activating factor acetylhydrolase (PAF-AH), an enzyme which inactivates PAF and their role during NEC in 2010. To study the role of the enzyme, genetically modified mice lacking PAF-AH were used to investigate their role in NEC using the HHF model. Deletion of PAF-AH reduced early mortality associated with bacterial load and asphyxia. However, the surviving mice had a significantly higher incidence of NEC and increased expression of pro-inflammatory mediators compared to wild-type mice. These results suggest that endogenous PAF-AH protects against NEC and that deficiency of this protein, which is characteristic of preterm infants, increases the risk of developing the disease (67).

Another study using the HHF model and examining PAF and its expression in different organs during NEC pathogenesis was conducted by Wang et al. In 2020. The study revealed that even though PAF is known to play a key role in the NEC inflammation process, inflammatory damage not only was seen within the intestine, but also outside of it in organs such as the lung, liver, and kidneys on different days during NEC induction. The degree and timing of inflammation, injury, and repair varied depending on the type of organ. However, overall it was described that organ repair was greatest on day 4 after NEC induction. In addition, secondary damage of varying degrees was found in the colon, terminal ileum, lung, liver and kidney, which was caused by inflammatory mediators originating from the NEC (68).

#### 3.1.4 Stem cells in NEC studied with the HHF model

Lastly, stem cell therapy involving in tissue repair and inhibiting inflammation seems to be a promising treatment option for NEC. Chen et al. also used the rat HHF model in 2020 to research the therapeutic effect of bone-marrow-derived mesenchymal stem cells (BM-MSCs) by enhancing their paracrine effect by silencing propyl hydroxylase 2 (PHD2). He used bone marrow-derived mesenchymal stem cells (BM-MSCs) with PHD2-knocked out and transferred it as PHDMSC-conditioned medium (PHDMSC-CM) into the rat model and investigated the effect of the medium on NEC. Summing up, Chen et al. demonstrated that treatment with PHDMSC-conditioned medium (PHDMSC-CM) resulted in less intestinal damage and thus reduced NEC incidences (69).

An Overview of the different animal models and methods used in the HHF can be found in the **Supplementary Table 1**.

### 3.2 Hypoxia formula (HF)

NEC induction using the hypoxia formula (HF) model is accomplished by exposing the subjects to hypoxia and formula feeding. Thus, in comparison to the HHF model, hypothermia is omitted. As a result, the HF model is considered to be less stressful for the subjects in the study. Using the HF method, different formula types, as well as LPS, with consequent bacterial colonization, can be used to induce NEC-like injury.

#### 3.2.1 Cells of the innate immune system studied with the HF model

With respect to the role of neutrophils in NEC pathogenesis, a 2018 study by Vincent et al. focused on the role of neutrophil extracellular traps (NETs) in NEC, revealing that the formation of NETs plays a crucial role in NEC pathogenesis. Through the inhibition of Protein Arginine Deiminase 4 (PAD4), a histone-modifying enzyme fostering chromatin decondensation and thereby NET formation, NEC manifestation was prevented. Thus, the study was able to show the importance of NETs formation in NEC pathogenesis in mice. Additionally, the study suggested that C57BL/6J mice may not be the most suitable animal model for NEC research with respect to neutrophils, as neutrophil levels in humans are more than twice as high as in C57BL/6J mice (49).

Therefore in 2020, in order to allow for a better NEC manifestation and neutrophil examination using the HF model, Klinke et al. developed a murine NEC model that resembles human neutrophil levels more closely. In this model, Granulocyte colony-stimulating factor (G-CSF) was administered to the test subjects to stimulate granulocyte production in the bone marrow, thereby increasing peripheral neutrophil levels (70).

In 2011, Maheshwari et al. also made use of the HF model to research the role of macrophages in NEC.The role of non-inflammatory macrophages was investigated in the context of incomplete differentiation in the preterm intestine and TGF-ß expression due the inflammatory process in NEC. In the human preterm intestine, macrophages exhibit an inflammatory cytokine profile. However, production of cytokines by macrophages in the developing intestine is suppressed by TGF-β, particularly the TGF-β2 isoform. Therefore, administration of TGF-β2 by enteral supplementation showed beneficial mitigation of experimental NEC in mice (71).

#### 3.2.2 Pattern recognition receptors studied with the HF model

The HF mouse model has been used extensively to study TLR4 signaling mechanisms.

In 2010, Sodhi et al. employed the HF model to research TLR4 and its inhibitory effect on enterocyte proliferation. The study group was able to demonstrate that activation of TLR4 seemed to impair proliferation of (1) enterocytes in the ileum of newborn mice and (2) intestinal epithelial cell 6 (IEC-6) enterocytes. As this effect has been shown to be mediated by activation of GSK3β and degradation of β-catenin, the investigators concluded that NEC is associated with a decrease in β-catenin expression and increase in mucosal GSK3β expression. In line with these findings, inhibition of TLR4 signaling is known to reverse inhibition of β-catenin signaling and as a result restores enterocyte proliferation (72).

Two years later, the same group focused on TLR4 and Notch signaling pathways, as well as their influence on goblet cell differentiation. It has been shown that absence or deletion of TLR4 can protect against NEC in mice. The study was able to demonstrate that protection against NEC is associated with enhanced differentiation of goblet cells in the small intestine, which in turn is mediated by suppressed Notch signaling. In the HF mouse model for NEC induction, TLR4 signaling via the Notch pathway has been shown to be increased in NEC, resulting in decreased numbers of goblet cells. Concluding, Sodhi et al. were able to prove that TLR4 expression affects goblet cell differentiation independently of the intestinal microbiome. Therefore, TLR4 signaling and the Notch pathway play a crucial role in the development of NEC and the regulation of goblet cell differentiation (73).

A more recent study focused on enteric glia loss and exaggerated TLR4 signaling during NEC. This study was able to demonstrate that loss of enteric glia in the preterm intestine contributes to the development of NEC, as mice lacking TLR4 on enteric glia were protected from NEC. In addition, neurotrophic factor (BDNF) released from enteric glia prevented TLR4 signaling and thus is proposed to possibly prevent NEC. Therefore, it is suggested that BDNF could be explored as a potential drug to treat NEC (74).

Not only the role of the TLR4 in NEC pathogenesis was studied with the HF model, but also the inhibition of TLR4 activation to reduce NEC. In 2012, NEC was induced via hypoxia and formula in neonatal mice. In this study they showed that amniotic fluid can inhibit TLR4 signaling within the intestine. They identified EGF as an important ligand in the amniotic fluid for the inhibition of TLR4 (36).

In 2021, Sodhi et al. focused on the human milk fucosyllactose (FL) oligosaccharides 2’-FL and 6’-SL. They directly bind to TLR4 and inhibited the activation of the receptor as shown in neonatal mice and piglets. NEC development was reduced in both HF animal models (26).

Counteracting TLR4’s promotion of NEC is NOD2. As such, Richardson et al. used a HF model to research the inhibitory effect of NOD2 on TLR4 during NEC pathogenesis in 2010. The results of this study suggest that NOD2 activation inhibits TLR4 expression in enterocytes and abrogates TLR4’s deleterious effects on intestinal mucosal injury and repair. Additionally, the activation of NOD2 seemed to reduce enterocyte apoptosis and attenuate the severity of NEC. Concluding, an inhibitory interaction between the TLR4 and the NOD2 signaling pathway in enterocytes seems to exist (75).

#### 3.2.3 Inflammatory mediators studied with the HF model

NF-κB and resulting inflammatory mediators have also been studied using the HF model.

The first study to be reviewed for this analysis is by Rentea et al., whose aim was to investigate early temporal expression of NF-κB in a neonatal rat model. Overall, the study was able to show that a rapid translocation and transcription of NF-κB shortly after initiation of enteral feeding takes place. In fact, an early inflammatory response was detected within the first hours after feeding, which is proposed to lead to persistent inflammation and potentially initiating the development of full-blown NEC. Summing up, NF-κB activation seems to play a key role in triggering inflammation in the early phase of NEC, thus leading to increased expression of proinflammatory proteins, before any detectable histological NEC damage (76).

In the same year oral administration of TGF-ß1, which is shown to inhibit NF-κB, was tested as a potential treatment for NEC. Here, oral administration of TGF-ß1 reduced the incidence of NEC through direct immunosuppressive effects on the intestinal epithelium due to its anti-inflammatory effects. Additionally, the effects of TGF-ß1 are associated with a decrease in pro-inflammatory mediators, such as interleukin-6 (IL-6) and IFN-γ (77).

With regards to interleukin influence on NEC, IL-22 and IL-12 were studied using the HF model. The effect of anti-inflammatory mediator IL-22 on NEC disease severity was assessed by Mihi et al. in 2021 through the exogenous administration of IL-22. Their results suggest that IL-22 administration protects the mucosal barrier and promotes regeneration of intestinal epithelial cells in mice with NEC. Even more, treatment with IL-22 did not affect the composition of the intestinal microbiome (78).

Regarding IL-12’s effect on NEC, Nadler et al. employed a rat HF model in 2000, as rats with NEC are considered to be more similar to human NEC patients. In rats that underwent the HF paradigm for NEC, a reduced IL-12 expression, as well as increased iNOS mRNA and enterocyte apoptosis in the intestinal ileum were found. As a result of peroxynitrile produced during NEC formation, enterocyte apoptosis and increased intestinal permeability were investigated (79).

#### 3.2.4 Components of the adaptive immune system studied with the HF model

The adaptive immune system is a defense system that provides specific and long-lasting protection against pathogens. It involves specialized cells like T and B cells, which recognize and respond to antigens. Through memory formation, it enables a faster and stronger response upon re-exposure to the same pathogen with antibodies, crucial for fighting infections and preventing disease recurrence.

Multiple studies have been conducted to assess components of the adaptive immune system on NEC pathogenesis using the HF model to induce NEC. The main focus of these studies is the overlying effect of regulatory T cells on NEC development.

Since it is known that NEC is associated with a low regulatory T cell (Tregs) to effector T cell (Teffs) ratio, Schulz et al. investigated the effect of HO-1 (heme oxygenase-1) on the T cell ratio using the HF mouse model for NEC induction. In this study, HET mice, a genetically diverse model, undergoing the HF NEC induction paradigm, demonstrated increased intestinal damage and a decreased ratio of Tregs to Teffs. Moreover, expression of genes involved in pattern recognition and neutrophil recruitment were increased in HET pups after NEC induction. Overall the study suggests that HO-1 modulates the ratio of Tregs found within the lamina propria during phases of inflammation and contributes to the protection of the intestine (80).

Another study focused on CCR9^+^ IL-17^+^ Treg cells, a subset of regulatory T cells that express both CCR9, a receptor, and IL-17 in both humans and mice with NEC. It was shown that mice and neonates with NEC had a higher proportion of CCR9^+^ CD4^+^ T cells in their peripheral blood than the control. The CCR9^+^ CD4^+^ T cells identified in the study were primarily CCR9^+^ IL-17-producing regulatory T cells (Tregs). The presence of IL-6 was found to promote the differentiation of CCR9^+^ Tregs into CCR9^+^ IL-17-producing Tregs, indicating a role for IL-6 in driving this polarization process. This suggests that IL-6-mediated polarization of CCR9^+^ Tregs into IL-17-producing Tregs contributes to the severity of NEC (81).

One year later, the same group studied the effect of melatonin administration on NEC in mice. Upon NEC induction and treatment with melatonin, it was shown that melatonin affects NEC by influencing the Th17/Treg balance in the intestine. More specifically, melatonin seems to block the differentiation of pathogenic Th17 cells while simultaneously causing an increase in the number of protective Tregs *in vitro* (82).

As shown in the previous section, the balance of Treg and inflammatory (Th17) CD4^+^ T cells (Treff) is of great importance in the development of NEC. The importance of this balance was also demonstrated by Egan et al. in 2015 by showing that TLR4 mediates increased polarization toward proinflammatory Treff cells and reduces Treg cells. This results in a shift of CD4^+^ Tregs toward a deleterious Th17 population leading to the severe intestinal inflammation characteristic of NEC (83).

The importance of a well-balanced lymphocyte concentration within the intestinal mucosa with respect to NEC was also the focus of the following study by Niño et al. in 2017. The authors hyposized that the administration of all-trans retinoic acid (ATRA) improves the incidence and severity of NEC by restoring the balance of T cells. The results of this study suggest, that ATRA modulates the imbalance between Treg and Treff and, as a result, promotes Treg expression. It significantly impairs proliferative capacity and mucosal healing. This lymphocyte balance seems to protect the intestinal crypt-based stem cells in mice by promoting the repopulation of these cells, while an increase in Th17 cells and a depletion of Tregs leads to increased apoptosis (7).

In 2007, Leaphart et al. focused on the relationship between enterocyte migration and IFN-γ. Key findings include that IFN-γ inhibits enterocyte migration by interfering with gap junction communication, particularly through connexin 43-mediated pathways that are critical for mucosal healing and enterocyte movement. Additionally, there appears to be a link between IFN release and decreased connexin 43 expression in NEC pathogenesis. In IFN^(-/-)^ mice it could therefore be shown that depletion of IFN led to restored connexin 43 expression and improved intestinal restitution (84).

An overview of the animal models and HF NEC induction paradigms are summarized in the **Supplementary Table 2**.

### 3.3 Formula feeding (FF)

The FF model for NEC induction was developed to study the influence of various formula feeds and at the same time have the possibility to spike them with different bacteria or products of bacteria.

#### 3.3.1 Cells of the innate immune system studied with the FF model

As it has been the case in the previous two models described, the FF NEC induction model has also been used to study macrophages and neutrophils.

One particular study induced NEC in mice via FF with the addition of *Cronobacter sakazakii* to the formula, a gram-negative bacterium which has been associated with clinical outbreaks of NEC. This model ultimately was used to explore the role of macrophages and neutrophils in the pathogenesis of NEC by depletion of these cells via Gr-1 antibody and carrageenan injection. Their absence leads to increased recruitment of dendritic cells (DCs), a higher bacterial load and increased production of pro-inflammatory cytokines. They also showed, that the depletion of neutrophils and macrophages in infected mice resulted in a severe inflammation of the intestine (85).

#### 3.3.2 Pattern recognition receptor studied with the FF model

In 2021, Kovler et al. used also a piglet FF model to research TLR4 mediated enteric glia loss during NEC development. The piglet model was used confirm the findings seen in mice. The outcomes of the study are summarized in the FF model section (74).

#### 3.3.3 Components of the adaptive immune system studied with the FF model

Using the FF model for NEC induction, the importance of dendritic cells in NEC pathogenesis was investigated by Emami et al. in 2011. This study showed that the interaction between the outer membrane protein A (OmpA)^+^ *Cronobacter sakazakii* and DCs plays a critical role in the development of NEC. In 2011, Emami et al. were able to demonstrate that a depletion of DCs in newborn mice protected against *Cronobacter sakazakii*-induced NEC, whereas transfer of DCs to DC-depleted mice restored susceptibility to NEC infection. The investigators hypothesized that the interaction between OmpA^+^ *Cronobacter sakazakii* and DCs leads to increased nitric oxide (NO) production in enterocytes, resulting in intestinal epithelial cell damage and apoptosis. Moreover, anti-inflammatory cytokines, such as TGF-β and IL-10, seem to also be involved in this process, with TGF-β causing an upregulation of inducible nitric oxide synthase (iNOS) resulting in epithelial damage (86).

Namachivayan et al. used a baboon model to study the inhibitory effects of Smad7 on the autocrine expression of TGF-β2 in intestinal epithelial cells in 2013. A downregulation of Smad7 resulted in in an increased TGF-β2 expression which leads to an exacerbation of NEC pathology (87).

In another study using the FF model for NEC induction preterm pigs were fed with parental nutrition. The authors focused on the interplay between TGF-β and LPS to regulate intestinal homeostasis via IL-8. The study was able to show that low levels of IL-8 may contribute to the maintenance and promotion of epithelial repair mechanisms. Paradoxically, high levels of IL-8 were also demonstrated to lead to inflammation (88).

Intraepithelial lymphocytes expressing the γδ T cell receptor (TCR) (γδ IEL) seem to also be important during NEC pathogenesis, as these cells have been shown to maintain intestinal integrity and prevent bacterial translocation in part by producing IL-17. More specifically, severe NEC-like intestinal injury was observed in TCRδ-deficient mice lacking γδ IEL cells, including CD8γδ IELs. In addition to these findings, a high TNFα, but low IL-17A expression was also observed. These results suggest that CD8 γδ IELs play a role in IL-17 production and gut barrier function early in life (89).

An overview of the animal models and FF NEC induction paradigms are summarized in the **Supplementary Table 3**.

### 3.4 TNBS model

It is known that the application of trinitrobenzene sulfonate (TNBS) elicits a strong inflammatory response of the intestine, as it is a nonspecific immunologic stimulant. The result of TNBS administration is increased chemotaxis and macrophage infiltration, leading to mucosal damage similar to the one seen in NEC.

In 2012, MohanKumar et al. used TNBS as a non-specific insult to induce NEC in mice. They compared the difference in NEC induction and their response to the insult between newborn and adult mice as well as the macrophage and leukocyte migration. The study concluded that intestinal injury is characterized by macrophage-rich leukocyte infiltration in neonatal mice whereas in adult mice, a pleomorphic leukocyte infiltration was observed (90).

The same group focused 2016 on the molecular mechanism of macrophage activation in NEC with focus on Smad7 in interrupting TGF-β signaling. In addition to the HHF model, MohanKumar et al. also used the TNBS model to investigate the role of macrophages during NEC as it has many advantages compared to the HHF model. They found an increased Smad7 expression in TNBS-induced NEC which interrupts TGF-β signaling and may promote inflammatory signaling (43).

One year later, the same group used this model to study the genetics of TNBS-mediated intestinal injury and human NEC. The results of the study confirmed similarities in the transcriptional profile between the TNBS-mediated mouse model and human NEC patients, as the TNBS-model activated nearly all the same biological pathways and transcriptional networks (44).

The overview of the TNBS models and NEC induction paradigms can be found in the **Supplementary Table 4**.

### 3.5 Paneth cell disruption (PCD) model

The PCD model was developed to mimic the decreased expression of Paneth cells found in human neonates with NEC. Paneth cells are critical regulators of the gut’s innate immunity and are part of the epithelial mucosal barrier. Moreover, they produce important antimicrobial peptides and regulate the innate immune system. As such, impaired Paneth cell function can create a proinflammatory state that is susceptible to injury. Moreover, Paneth cells also regulate the intestinal bacterial composition (91). To understand whether Paneth cell disruption plays a role in NEC pathogenesis, Zhang et al. developed a model in which Paneth cells undergo ablation via dithizone or diphtheria toxin injection (42).

#### 3.5.1 Innate immune cells studied with the PCD model

In 2017, White et al. published a study on the development of NEC associated with Paneth cells. They showed that disruption of Paneth cells in mice leads to NEC when exposed to live bacteria, and that this occurs independently of TLR4 signaling. The study highlights the critical role of Paneth cells in the pathogenesis of NEC and shows that their disruption, in combination with exposure to bacteria, causes intestinal damage that resembles human NEC. Importantly, their research highlights that live bacteria are necessary for the initiation of this damage, whereas TLR4 activation is not required for the development of NEC in this context (45).

Lueschow et al. focused on the role of Paneth cells in the context of the immature intestinal microbiome in 2018. The results of their study suggested that Paneth cell depletion of the immature small intestine in mice induces significant changes in the composition of the intestinal microbiota. Additionally, the depletion of Paneth cells followed by a Klebsiella-induced dysbiosis was shown to induce a phenotype similar to the microbiota of human neonates with NEC. Based on the findings of this study, it can be assumed, that Paneth cells play a key role in NEC pathogenesis (46).

Another study using the PCD model conducted in 2021 by Chaaban et al. used CD-1 mice investigated the presence of NETs and nucleosomes in human and mouse tissue. Additionally, the effects of chloramidine and pan-PAD, both for NETs inhibition, were examined and demonstrated following results: (1) increased nucleosome levels in mice and human NEC tissue, and (2) an effective inhibition of pan-PAD in the realms of NETs formation during NEC (92).

For the PCD model, an overview can be found in the **Supplementary Table 5**.

### 3.6 Dextran sodium sulfate (DSS) model

One of the newer models to study NEC is the DSS model, in which administration of DSS results in NEC-like lesions with humoral and cellular immune responses throughout the intestine.

#### 3.6.1 Inflammatory mediators studied with the DSS model

One study examining the expression of the chemokine CXCL2 during the inflammatory process in NEC was investigated using the DSS model, while simultaneously comparing adult with neonatal mice. The authors reported colonic inflammation with increased expression of CXCL2 mRNA in adult mice treated with DSS. In contrast, neonatal mice treated with either (1) DSS or (2) LPS in combination with hypoxia/hypothermia exhibited tissue inflammation in both the colon and small intestine. Here, the DSS model yielded increased intestinal expression of CXCL2 mRNA. What is more, LPS administration primarily triggered local recruitment of neutrophils while DSS-treated mice showed increased infiltration of monocytes/macrophages. These results indicate that DSS has the potential to induce NEC-like lesions (47).

A summary of the DSS model and the NEC induction paradigms can be found in the **Supplementary Table 6**.

### 3.7 Organoids as a NEC model – NEC in a dish

Organoids are 3D-cell cultures, which can be used to represent particular organs and study their functions. Organoids can be grown using different tissue entities as well as different species. Using organoids to study NEC is relatively new. However, both human and murine organoid models have been developed for NEC research.

Adding to the vast opportunities to study NEC in a dish is the establishment of a reversed enteroid polarity – also known as an apical-out enteroid – model, resulting in the apical surface facing outward (93). This organoid model has been validated by Liebe et al. in 2023. The benefit of using an apical enteroid model is the direct access to the laminal surface. This allows for a more in depth analysis of pathogen interactions with the intestinal epithelial barrier (94).

#### 3.7.1 Inflammatory mediators studied with the organoid model

To study necroptosis in NEC, Werts et al. evaluated human NEC tissue, an NEC mouse model, and a *ex vivo* NEC model using mouse intestinal organois as well as differentiated and undifferentiated mouse organoids in the presence of NEC bacteria and hypoxia to induce NEC. In the context of necroptosis, the protective effects of human breast milk and its associated milk oligosaccharides were examined using the organoid model. Furthermore, the inflammatory effect and the induction of necroptosis by TLR4 activation were studied using this model. The study shows that necroptosis, a form of programmed cell death, plays an important role in the development of NEC. Activation of necroptosis is observed in the intestinal epithelium of both humans and mice with NEC. Inhibition of necroptosis reduces NEC pathology in mice, and its induction is particularly pronounced in the differentiated epithelium in the organoid model. Breast milk and its component 2’-fucosyllactose (2’FL) reduce necroptosis in the *ex vivo* NEC model (95).

A newer study focused on IL-22 as potential treatment option for NEC. IL-22 is a key modulator of intestinal microbial communities and can control intestinal homeostasis by preventing harmful inflammatory responses in adults, where the protective effect could be demonstrated. However, the role of IL-22 in preterms is still unknown. Here, enteroids were used to compare different genetic expressions induced by IL-22 from human NEC and healthy tissue enteroids with the expression in a NEC-mouse model with induced NEC. Both, mice and human enteroids were treated with IL-22, resulting in similar results: It triggers an anti-microbial response by upregulating genes such as *Retnlb*, *Reg3g*, *Fut2*, and *S100* genes, involved in the regulation of the microbiome. IL-22 administration seemed to protect the mucosal barrier and promoted the regeneration of intestinal epithelial cells (78).

### 3.8 NEC on a chip

An intestine-on-a-chip has been described as an improved preclinical model to study NEC, as it allows a comprehensive analysis of the pathophysiology of NEC. In this model, unlike the NEC-in-a-dish model, epithelial cells were seeded onto a microfluidic device to form a monolayer. This model utilizes a combination of healthy and NEC enteroids co cultured with human intestinal microvascular endothelial cells and patient-derived microbiota. In 2023, Lanik et al. used this advanced model to assess the interactions between intestinal epithelium, endothelium, and the microbiome in preterm infants. They showed an upregulation of different genes involved in ferroptosis, and necroptosis pathway compared with the healthy control (96).

Both models, NEC-in-a-dish and NEC-on-a-chip are summarized in the **Supplementary Table 7**.

## 4 Conclusion

Necrotizing enterocolitis (NEC) is one of the most devasting diseases affecting premature neonates. As it involves the interaction of various inflammatory components, ultimately leading to tissue damage and necrosis in the intestine, various models must be used to characterize the disease. However, the usage of animal models bears many limitations, such as differences in immune systems or a distinctive development of the intestine. This makes translation of results from animal NEC studies to humans difficult. However, recently translational models like organoids and NEC on a chip have been developed, which are very promising in order to study NEC. These models have been shown to replicate physiological, immunological, and pathological aspects of NEC. What is more, as they are derived from human or animal tissue, the usage of animals to study NEC can be reduced. As organoids can mimic relatively complex interactions within the intestinal tissue and immune cells involved in NEC, animal models might potentially be obsolete in the future.

## Author contributions

LB: Writing – original draft, Writing – review & editing. DV: Writing – review & editing. MB: Conceptualization, Supervision, Writing – review & editing. JK: Supervision, Writing – review & editing.
