# Supplementary material for: Immunological aspects of necrotizing enterocolitis models: a review
Source: Front Immunol. 2024 Jul 22;15:1434281. doi: 10.3389/fimmu.2024.1434281 (PMC11298363; doi:10.3389/fimmu.2024.1434281)
Supplement: Supplementary file 1 [file Table_1.docx]

Supplementary Material

Immunological aspects of necrotizing enterocolitis models: a review

Laura Blum^1^, Deirdre Vincent M.D. ^1^, Michael Boettcher M.D. Ph.D.^1^, Jasmin Knopf Ph.D.^1,^*****

Department of Pediatric Surgery, University Medical Center Mannheim, University Heidelberg, Theodor-Kutzner-Ufer 1-3, 68167 Mannheim, Germany

*** Correspondence:**Dr. rer. nat. Jasmin Knopf

Department of Pediatric Surgery, University Medical Center Mannheim, University Heidelberg, Theodor-Kutzer-Ufer 1-3, 68167 Mannheim

jasmin.knopf@medma.uni-heidelberg.de

0621/838-9049

# Supplementary Data

Table 1: Overview of the experimental setup to study immunological factors in NEC with the HHF model.

| Immunological Aspect | Year | Model | | Sample Size/groups | | NEC induction | Ref. |
| --- | --- | --- | --- | --- | --- | --- | --- |
| Macrophages | 2016 | C57BL/6 mouse pups | | Different experiments | | Hypoxia:(99% N_2_) 1 min, 2x  Hypothermia: 4°C, 10 min, 2x  Formula: Gavage-feeding, instant formula, every 3h | [43] |
|  | 2021 | C57BL/6 mouse pups | | 4 Groups: control  NEC,  NEC+NS (normal saline)  NEC+liposomal clodronate (CL)  n=48 | | Hypoxia: (95% nitrogen), 10 min, 3x daily, every 4h  Hypothermia: 4°C, 10 min, 3x daily, every 4h  Formula: breastfed | [52] |
| TLR4 | 2006 | C3H/HeJ  C3HeB/FeJ  mice | | Different experiments | | Hypoxia: (100% nitrogen), 1 min, 2x daily  Hypothermia: 4°C , 10 min, 2x daily  Formula: puppy formula | [53] |
|  |  | Sprague-Dawley rats | | Different experiments | | Hypoxia: (100% nitrogen), 1min,  Hypothermia: 4°C , 10 min, 2x daily  Formula: puppy formula, every 3h, orogastric feeding catheter |  |
| TLR4  TLR2  NOD2 | 2007 | Wistar rats | | Group 1: full term, mother-fed, n = 10  group 2: mother-fed n = 14  group 3: hand-gavage fed with a protein rich formula after a hypoxia–reoxygenation procedure n = 10 | | Hypoxia: 100% CO_2,_ 5 min.  Hypothermia:  Formula: Gavage feeding every 4 hours for 48 hours. Hypoxia (100% CO_2_) for 5 minutes, exposure to cold (1°C), followed by reoxygenation with (100% O_2_) for 5 minutes. | [54] |
| TLR2  TLR4 | 2014 | Sprague–Dawley rats | | Gln intervention group n = 20  control group n= 20 | | Hypoxia: Nitrogen 10 ml/min  Hypothermia: 4°C, 10 min  Formula: formula milk, every 4 h, formula after 24 h | [55] |
| TLR4  TLR9 | 2017 | BALB/c mice (specific pathogen‑free level) | | Control group n=25  test group, n=25 | | Hypoxia: Nitrogen 10 ml/min  , 90 sec  Hypothermia: 4°C, 10 min  Formula: mouse milk substitutes | [56] |
| TLR4 | 2022 | C57BL/6J mice  TLR4 KO mice | | Different experiments | | Hypoxia: 5% oxygen and 95% nitrogen, 3 min  Hypothermia: 4°C, 10 min  Formula: Formula every 4 h  Twice a day, 72 h. | [57] |
| TLR4 | 2022 | Sprague Dawley rats | | 4 groups  n=12 per group | | Hypoxia: 95% nitrogen, 90 sec.  Hypothermia: 4 °C, 10 min, every 12 h  Formula: Formula, 6x per day | [58] |
| TLR4  NOD2 | 2023 | Sprague-Dawley rats | | 3 groups  normal group NEC group  NEC + Astaxanthin group  n =8 each group | | Hypoxia: 100% nitrogen  Hypothermia: 4 °C, 10  Formula: Rat milk  2x daily, 72 h . | [59] |
| IL-6 | 2021 | Sprague-Dawley rats | 3 group  NEC + placebo  NEC + tocilizumab  control group | | Hypoxia: 100% CO2 for 10 min, 97% O_2,_ 5 min  Hypothermia: + 4 ◦C, 5 min  Formula: Formula every 3 h  2x daily, 3 days | | [60] |
| IL-10 | 2012 | Sprague–Dawley rats | Different experiments | | Hypoxia: 5% O_2_, 10 min  Hypothermia: 4°C, 8 min  Formula: 3x daily | | [61] |
|  |  | C57Bl/J and congenic IL-10−/− mice | Different experiments | | Hypoxia: 1 min at 0% O_2_  Hypothermia: 10 min at 8°C  Formula: Formula every 3 h | |  |
| TNFα | 2005 | Sprague-Dawley rats | 3 groups:  group 1 (n = 20), formula feeding, asphyxia, cold exposure  group 2 (n = 9), treat as group 1 + TNF-a antibody intraperitoneally group 3 (n = 17), dam-fed | | Hypoxia: Breathing 100% nitrogen for 60 seconds  Hypothermia: 4°C, 10 min  Formula: Rat milk every 3 h. | | [62] |
|  | 2014 | Wistar albino rats | 3 groups  Control n=10  NEC+Placebo n=7  NEC+Etanercept n=10 | | Hypoxia: pre-perfused with 100 % CO_2_ for 10 min, 97 % O_2_ for 10 min  Hypothermia: 4 °C for 5 min  Formula: Special rodent formula  Twice daily. | | [63] |
|  | 2016 | Sprague-Dawley rats | 3 groups  NEC  NEC+infliximab, and control | | Hypoxia: 100% CO_2_ inhalation, 10 min, 97% O_2,_ 5 min  Hypothermia: 4◦C cold for 5 min Formula: Special rodent formula with puppy-canine milk, every 3 h  Twice daily, 72 h. | | [64] |
| NF-κB | 2007 | Sprague-Dawley rats | Group without bacteria n=18  group with bacterial inoculation n=15 | | Hypoxia: 60 s in 100% N_2_  Hypothermia: 4°C, 10 min  Formula: Colony forming unit (CFU) of a standardized bacterial mixture and formula. | | [65] |
|  | 2019 | Cx3cr1GFP/þ  C57BL/6J  LysmCre/þ IKKbf/f, Villin Creþ/ IKKbf/f, and IKKbf/f  mice | Different experiments | | Hypoxia: 60 seconds in 100% N_2_  Hypothermia: 4°C, 10 min  Formula: CFU (murine adult bacteria) with LPS, every 3 h.  Twice daily. | | [66] |
| PAF | 2010 | C57BL/6J PAF-AH knockouts mice | Different experiments | | Hypoxia: 60 s, 100% N_2_  Hypothermia: 4°C, 10 minutes  Formula: Formula with Serratia marcescens and Streptococcus viridans. | | [67] |
|  | 2020 | Sprague Dawley rats | NEC group  control group  n=70 | | Hypoxia: 100% N_2_ for 90 s  Hypothermia: 4 °C, 10 min  Formula: Artificial formula and dairy substitutes  2x daily, 72 h. | | [68] |
| Stem cells | 2020 | Sprague Dawley rats | n=58 | | | Hypoxia: 100% N_2_ for 50 s  Hypothermia: 4 °C, 10 min  Formula: Hypercaloric formula  2x daily | [69] |

Table 2: Overview of the experimental setup to study immunological factors in NEC with the HF model.

| **Immunological**  **Aspect** | **Year** | **Model** | Sample Size/Groups | **NEC induction** | **Ref.** |
| --- | --- | --- | --- | --- | --- |
| Neutrophils | 2018 | C57BL/6J mice | n=76 | Hypoxia: 10 min, 5% oxygen  Formula: Neocate and LPS  2x daily, 6 days | [49] |
|  | 2020 | C57BL/6 ELANE-KO mice | n=44 | Hypoxia: 10 min, 5 % oxygen  Formula: Neocate and LPS  3x daily, 4 days | [70] |
| Macrophages | 2011 | DNIIR mice  Treated with zinc sulfate | Different experiments | Hypoxia: 5 % oxygen for 2 min  Formula: Formula, every 3 h.  2x daily | [71] |
| TLR4 | 2010 | C3H/HeOUJ  C3H/HeJ mice | Different experiments | Hypoxia: 5 % O_2,_ 95 % N_2,_ 10 min  Formula: Fromula, 5x per day  2x daily, 4 days | [72] |
|  | 2012 | Tamoxifen treated villin-Cre-ERT2 | Different experiments | Hypoxia: 5 % O_2,_ 95 % N_2,_ 10 min  Formula: Fromula, 5x per day  LPS i.p., 2x daily, 4 days, Amniotic pretreatment | [36] |
|  | 2021 | C57BL/6J mice | Different experiments | Hypoxia: 5 % O_2,_ 95 % N_2,_ 10 min  Formula: Fromula + bacteria, 5x per day, Fromula +2´FL/6´FL  2x daily, 4 days. | [26] |
|  | 2012 | TLR4^ΔIEC^ mice  TLR4^del/+^ TLR4^+/−^ mice | Different experiments | Hypoxia: 5 % O_2,_ 95 % N_2,_ 10 min  Formula: Fromula, 5x per day  2x daily, 4 days. | [73] |
|  | 2021 | Sox10-TLR4^ko^ mice  Glia-deficient mice (Plp1^ΔDTR^,Sox10^ΔDTR^, Bdnf^ΔDTR^) | 6–8 animals per group | Hypoxia: 5% O_2_ to 95% N_2,_ 10 min  Formula: Advance infant formula supplemented with enteric bacteria, 5x per day | [74] |
| NOD2 | 2010 | TLR4−/− mice  C57Bl-6 mice  NOD2−/− mice | five mice/group | Hypoxia: 5 % O_2_, 95 % N_2_, 10 min  Formula: Fromula, 5x per day  2x daily, 4 days. | [75] |
| NF-κB | 2013 | Sprague-Dawley rat | Different experiments | Hypoxia: 3x daily  Formula: Formula with LPS, every 4 h | [76] |
| TGFß | 2013 | Sprague-Dawley rat | dam-fed control group (n=20)  experimental NEC group (n=48) | Hypoxia: 5 % oxygen and 95 % nitrogen for 10 min  Formula: CFU of Serratia marcescens, Klebsiella pneumoniae, and Streptococcus viridans with formula, every 3 h  3x daily | [77] |
| IL-22 | 2021 | C57BL/6 and villin cre mice  IL22ra1^fl/fl^ and IL22^−/−^ mice | Different experiments | Hypoxia: 95% N_2,_ 5% O_2,_ 10 min  Formula: Formula with LPS + human bacteria  6x daily  2x daily, 72h | [78] |
| IL-12 | 2000 | Sprague–Dawley rats | Groups 1(n=8) and 2 (n=9): breast-fed,  Groups 3 (n=12) and 4 (n=14): formula feeding Groups 2 and 4 + hypoxia | Hypoxia: 5% O_2_, 95% N_2,_ 3 min  Formula: special rodent formula, 3x daily | [79] |
| Tcells | 2015 | HO-1KO mice | Control: breastfeed  HO-1 deficiency model  n=51 total | Hypoxia:  Formula:  Feed of heme by oral gavage 24 and 3 h prior, formula feedings for 78 h with a total of five 2-min hypoxic exposures. | [80] |
|  | 2016 | C57BL/6  Rag1–/–  IL-17GFP  RoRγt-GFP  TLR4^ΔIEC^  TLR4^IEC–OVER^ mice | Different experiments | Hypoxia: 5% O_2_  Formula: Enteral formula+ enteric bacteria | [83] |
|  | 2019 | C57BL/6 mice | 3 groups  dam-fed;  NEC induction; NEC induction + anti-IL6R supplement.  n=83 total | Hypoxia: 5% O_2_, 95% N_2,_ 10 min  Formula: Formula+ enteric bacteria  2x daily | [81] |
|  | 2020 | C57BL/6 mice | 4 groups  dam-fed;  NEC induction; NEC induction + melatonin supplement; NEC induction + saline supplement; NEC induction + melatonin supplement + Treg ablation; NEC induction + saline supplement + Treg ablation.  n=60 total | Hypoxia: 5% O_2_, 95% N_2,_ 10 min  Formula: Formula+ enteric bacteria  2x daily | [82] |
|  | 2017 | C57Bl/6  Foxp3^+DTR^ mice | n=120 | Hypoxia: 5% O_2_, 95% N_2,_ 10 min  Formula: Enteral formula, 5x daily, 4 days | [7] |
| INFy | 2007 | IFN-/- mice |  | Hypoxia: 5% oxygen for 1 min  Formula: Enteral formula  2x daily, 4 days | [84] |

Table 3: Overview of the experimental setup to study immunological factors in NEC with the FF model.

| **Immunological**  **Aspect** | **Year** | **Model** | Sample Size/Groups | | **NEC induction** | **Ref.** |
| --- | --- | --- | --- | --- | --- | --- |
| Macrophages/  Neutrophils | 2012 | C57BL/6 mice | Six independent experiments, 15 animals per group. | | Fed of 10^3^ CFU of CS in 10 μl of sterile PBS orally | [85] |
| TLR | 2021 | White Yorkshire piglet | Different experiments | | Gavage formula every 3h for 4 days supplemented with enteric bacteria made from an infant with surgical NEC | [74] |
| Dendritic  cells | 2011 | C57BL/6 mice | Different experiments | Formula feeding with colony forming units OmpA+/- and *Cronobacter sakazakii* | | [86] |
| TGFß | 2013 | Olive/yellow baboons (Papio cynocephalus anubis/P. cynocephalus cynocephalus) | NEC animals  n=7 | Parenteral nutrition after 24 h. Enteral feeds after day 5 using a primate or human infant formula | | [87] |
|  | 2014 | Danish Landrace × Large White × Duroc piglet | bovine colostrum (COLOS, n=27)  infant formula (IF, n=27) | Fed with parenteral nutrition with or without minimal enteral nutrition for 2 days followed by 2 days of full enteral nutrition | | [88] |
| IEL | 2014 | C57BL/6J TCRδ-deficient (tcrδ^-/-^) mice | Different experiments | Injection with platelet activating factor, E. coli, and LPS intraperitoneally | | [89] |

Table 4: Summary of TNBS NEC induction in order to study the role of macrophages in NEC.

| **Immunological**  **Aspect** | **Year** | **Model** | **Sample Size/Groups** | **NEC induction** | **Ref.** |
| --- | --- | --- | --- | --- | --- |
| Macrophages | 2012 | C57BL/6 mice | Different experiments | TNBS administration | [90] |
|  | 2016 | C57BL/6 mice | n=15 | TNBS administration gavage and rectal instillation | [43] |
|  | 2017 | C57BL/6 mice | n=11 |  | [44] |

Table 5: Overview of NEC studies using the DK/PCD model to study innate immune cells involved in NEC.

| **Immunological**  **Aspect** | **Year** | **Model** | **Sample Size/Groups** | **NEC induction** | **Ref.** |
| --- | --- | --- | --- | --- | --- |
| Paneth cells | 2012 | CD-1 mice | Different experiments | Dithizone in Li_2_CO_3_, or 6 hours after injection, enterally infected by gastric gavage with 1×10^11^ CFU Klebsiella | [42] |
|  | 2017 | C57BL/6J  PC-DTR mice  TLR4−/− mice | Different experiments | Dithizone-induced Paneth cell depletion: dithizone dissolved in a NH_4_OH/EtOH solution, intraperitoneal injected and afterwards fed with colony forming units.  Diphtheria toxin-induced Paneth cell depletion: injected with diphtheria toxin in phosphate buffered saline and 24 h, mice were gavage-fed with colony forming units | [45] |
|  | 2018 | C57BL/6J  PC-DTR mice  Rosa mice | Different experiments |  | [46] |
| Neutrophils | 2021 | CD-1 mice | sham (n = 10),  NEC (n = 20)  NEC + Clamidine (n = 20). | Injection of dithizone in Li_2_CO_3_, enteral gavage of live Klebsiella pneumoniae | [92] |

Table 6: Summary of DSS NEC induction to study inflammatory mediators.

| **Immunological**  **Aspect** | **Year** | **Model** | **Sample Size/Groups** | **NEC induction** | **Ref.** |
| --- | --- | --- | --- | --- | --- |
| CXCL2 | 2017 | C57BL/6J mice | First experiment:  DSS n=10, LPS n=10, controls n=9  Second experiment:  DSS n=8, LPS n=8, controls n=8 | Adult mice: 3% (w/v) DSS added to the drinking water.  Neonatal mice: Formula feeding supplemented with 3% DSS without physical stress | [47] |

Table 7: Overview of the different in-vitro models.

| **Model** | **Advantage** | **Limitation** | **How to overcome the limitations?** | **Ref.** |
| --- | --- | --- | --- | --- |
| NEC in a dish | - Standardized protocols - large-scale-approach - same genetic background as human - use of fresh or frozen tissue | - Lack of immune components of the human intestine, circulating factors | - Development of organoid co-culture s | [78, 94, 95] |
| NEC on a chip | - Inoculated with enteric bacteria - visible 3D-villus-like structure | - Lack of immune cells, circulating factors - aerobic conditions (anaerobic condition in the intestine) | - Co-culture on-a-chip - built of anaerobic conditions | [96] |
